# Thyroid cancer risks among medical radiation workers in South Korea, 1996–2015

**DOI:** 10.1186/s12940-019-0460-z

**Published:** 2019-03-11

**Authors:** Won Jin Lee, Dale L. Preston, Eun Shil Cha, Seulki Ko, Hyeyeun Lim

**Affiliations:** 1 Department of Preventive Medicine, Korea University College of Medicine, 73, Goryeodae-ro, Seongbuk-gu, Seoul, 02841 South Korea; 2 Hirosoft International, Eureka, CA USA; 3 Environmental Health Center, Asan Medical Center, Seoul, South Korea

**Keywords:** Cohort, Neoplasm, Occupational exposure, Radiation, Thyroid gland, Workers

## Abstract

**Background:**

Thyroid cancer rates, especially among children, are known to be increased by radiation exposure. However, little is known about the impact of chronic low-dose radiation exposure on thyroid cancer risk in adulthood. This study examined radiation effects on thyroid cancer rates as well as an overall evaluation of thyroid cancer risk among medical radiation workers.

**Methods:**

Data on all diagnostic medical radiation workers enrolled in the national dosimetry registry between 1996 and 2011 were linked with the cancer registry data through 2015. Standardized incidence ratios (SIRs) were used to compare the observed cancer incidence rates in this population to those for the general population while internal comparisons were used to estimate relative risks (RRs) for occupational history and excess relative risks (ERRs) were used to quantify the radiation dose-response relationship.

**Results:**

Overall, 827 thyroid cancer cases were reported among 93,922 medical radiation workers. Thyroid cancer SIRs were significantly higher than expected for both men (SIR 1.72, 95% confidence interval [CI] 1.53 to 1.91) and women (SIR 1.18, 95% CI 1.08 to 1.28). However, RRs for thyroid cancer by job title and duration of employment showed no particular pattern among diagnostic medical radiation workers. There were no indications of a significant dose effect on thyroid cancer rates for either men (ERR/100 mGy 0.07, 95% CI -0.38 to 0.53) or women (ERR/100 mGy -0.13, 95% CI -0.49 to 0.23). The findings were similar for different job titles or when limited to workers employed for at least one year.

**Conclusions:**

While thyroid cancer incidence rates among Korean medical radiation workers were somewhat higher than those in the general population, there was no significant evidence that this increase was associated with occupational radiation dose. Additional follow-up together with consideration of other risk factors should provide useful information on thyroid cancer rates in this cohort.

**Electronic supplementary material:**

The online version of this article (10.1186/s12940-019-0460-z) contains supplementary material, which is available to authorized users.

## Background

In recent decades, a worldwide increase in thyroid cancer incidence rates has been observed [1]. While overdiagnosis due to improved access to healthcare and screening is seen as an important contributor to this increase [2, 3], it has been suggested that demographic, environmental, and other factors have contributed to this increase [4, 5]. With evidence provided by studies of atomic bomb survivors, medically exposed children, or populations reported to have been acutely exposed to high-dose irradiation, ionizing radiation is the only established environmental carcinogen for thyroid cancer [6]. While radiation exposure in adulthood is associated with lower risk of radiation-induced thyroid cancer than that in children [7], occupational exposures to man-made sources of radiation in the medical and industrial field increase yearly [8], and some reports have linked occupational radiation exposure to thyroid cancer risk [9, 10].

Medical radiation workers, many of whom are women, are the largest group of workers with occupational radiation exposures and their number is rapidly increasing worldwide due to the increasing use of modern medical practices [8]. These workers comprise an identifiable professionally certified population with routinely obtained information on radiation exposure. In addition, while doses received by medical radiation workers may exceed those received by the general population, the nature of the exposures is generally qualitatively similar to those received by the general population. Cohort studies of medical radiation workers have reported radiation-associated excess risks of leukemia, skin cancer, and breast cancer in workers employed before 1950 [11]. However, the findings for thyroid cancer were limited by the small number of cases and, in most studies, the lack of thyroid organ doses.

We constructed a registry-based cohort by combining information on all diagnostic medical radiation workers enrolled in the South Korean National Dose Registry (NDR) between 1996 and 2011 with national mortality data with coverage through the end of 2015. A general description of mortality in this cohort was reported recently [12]. A historical dose reconstruction was performed [13] and organ-specific radiation doses were estimated for the cohort [14]. We have extended this study by linking to data on cancer incidence in order to investigate the role of occupational radiation exposure in cancer development. In this report, we compare thyroid cancer rates among South Korean diagnostic medical radiation workers to those seen in the general population and examine the evidence for effects of occupational characteristics and radiation dose on thyroid cancer risk in the cohort.

## Methods

### Study population

Since 1996, the Korea Center for Disease Control and Prevention (KCDC) has maintained a centralized national dose registry and created a lifelong follow-up management system for radiation dose for all medical radiation workers involved in diagnostic radiology [15]. The study population comprised 94,396 diagnostic radiation workers enrolled in the NDR between 1996 and 2011. The cohort includes radiologists (*n* = 1718), physicians (*n* = 18,186), dentists (*n* = 15,552), dental hygienists (*n* = 12,988), radiologic technologists (*n* = 25,969), nurses (*n* = 7218), and other medical assistants (*n* = 12,765). Medical workers involved with nuclear medicine and therapeutic departments are under the Nuclear Safety and Security Commission and thus were not included in this system. Registry information included name, gender, personal identification number, occupational group**,** quarterly dose data, and the beginning and end of the period of measurement. Among workers in the database, those with any cancer prior to enrollment (*n* = 462) or who had invalid data (*n* = 12), were excluded; thus, 93,922 workers were included in the analysis. For some sensitivity analyses, the 13,439 cohort workers who were employed for less than one year were excluded. The present study was reviewed and approved by the Institutional Review Board of Korea University (1040548-KU-IRB-16-203-A-1).

### Ascertainment of cancer incidence and vital status

Cancer incidence was ascertained by linkage to the Korean Central Cancer Registry (KCCR), the centralized national registry from the Korean National Cancer Center, upon our request. We sent the personal identification numbers of study subjects to the KCCR and KCCR staff used this information to link to the cancer incidence data. This linkage method is thought to be accurate and largely complete because of the uniqueness of the personal identification numbers of individuals in South Korea and the completeness of the KCCR data (98.2% in 2015) [16]. KCCR provides data regarding cancer codes (International Classification of Diseases and Related Health Problems, 10th Revision [ICD-10]) and International Classification of Diseases for Oncology, 3rd Edition [ICD-O-3]); site; histologic types; Surveillance, Epidemiology, and End Results (SEER) summary stage; and the date of diagnosis for all cancers linked to cohort members. Histological subtypes of thyroid cancer were classified according to the ICD-O-3 as papillary carcinoma, follicular carcinoma, medullary carcinoma, and others [17]. Vital status was ascertained by Statistics Korea using a linkage method like those used for the KCCR linkage.

### Thyroid dose calculation

Badge dose measurements, using a personal thermoluminescent dosimeter, were collected quarterly by the personnel monitoring centers designated by the KCDC. After reconstructing radiation doses for all diagnostic radiation workers using personal badge doses and survey data [13], we estimated radiation organ doses by considering relevant uncertainty factors such as apron usage, badge location, dominant energy of the diagnostic radiation fields [14], used in the dose reconstruction for the US radiologic technologists study [18, 19]. However, unlike the US radiologic technologists dosimetry, which provides multiple realizations each annual dose, our current dosimetry is a deterministic system that provides only a single estimate for each annual dose. Briefly, the annual and cumulative individual badge doses based on H_p_ (10) (dose at a tissue depth of 10 mm from the dosimeter) were calculated by combining the quarterly badge readings of the workers enrolled in the NDR. The lowest detectable quarterly level of the national dosimetry registry, 0.01 mSv, were taken as 0.005 mSv to minimize bias in the estimated doses. Historical badge doses were reconstructed for workers who began working with radiation before 1996 (*n* = 13,144; 14.0% of the total enrollees in the NDR), using an annual dose model that describes doses as a log-linear function of calendar year and age at the year of exposure [13]. Organ-specific doses were estimated as the product of the individual cumulative badge doses and two conversion coefficients provided by the International Commission on Radiological Protection (ICRP): the organ-absorbed dose per unit of air kerma free-in-air [20] and the personal dose equivalent per unit of air kerma free-in air [21]. The equation was ${H}_p(d)\left[\frac{D_T}{K_a}/\frac{H_p(d)}{K_a}\right]$, where *H*_*p*_(*d*) is the equivalent dose; $\frac{D_T}{K_a}$ is the air kerma-to-organ dose conversion coefficient; and $\frac{H_p(d)}{K_a}$ is the air kerma-to-personal dose equivalent conversion coefficient. We assumed that the most common irradiation geometry was anterior to posterior and under the present assumptions that the predominant energy of diagnostic radiation fields is between 30 and 40 keV (i.e., the conversion coefficients were 0.88 for males and 0.95 for females). To account for the effects of apron usage and badge location, we considered the effect of probability factors of wearing an apron, badge position (i.e., inside or outside the apron), and apron attenuation factors. We assumed an 80% reduction in exposure beneath an apron based on a previous Korean study [22]. Incorporating the personal badge doses, conversion coefficients, probabilities of wearing an apron, and badge locations, the formula for the organs above the apron (i.e., thyroid organ) is: *D*_*o*_ = *D*_*c*_ ∗ *R*_*coef*_ ∗ (*P*_*NoA*_ + *P*_*AO*_ + *P*_*AU*_/*AA*), where *D*_*o*_ is the organ dose; *D*_*c*_ is the personal cumulative badge dose; *R*_*coef*_ is the conversion coefficient at mean value between 30 and 40 keV in the anterior to posterior direction of exposure; *P*_*NoA*_ is the probability of not wearing aprons at work; *P*_*AO*_ is the probability of wearing aprons with the badge outside; *P*_*AU*_ is the probability of wearing aprons with the badge inside; and *AA* is the apron attenuation factor.

### Statistical analysis

Each individual contributed person-years at risk from 1996 or from the year of start of work based on the NDR, whichever occurred later. The end of follow-up was taken as the earliest of the following: the date of thyroid or other cancer diagnosis, the date of death, or December 31, 2015. The DATAB module in Epicure (Risk Sciences International, version 2.0, Ottawa, Canada) was used to create a person-year table stratified by sex, attained age (< 25, 5-year intervals from age 25 to 84, ≥85 years), calendar time (< 2000, 2000–2004, 2005–2009, ≥2010), year of birth (< 1960, 1960–1969, 1970–1979, ≥1980), year of entry (< 2000, 2000–2004, ≥2005), job titles (seven categories, as described above), and cumulative dose (< 0.5, 0.5–1.0, 1.0–2.5, 2.5–5.0, 5.0–10.0, 10.0–20.0, ≥20 mSv). The expected number of incident thyroid cancers for each cell was computed as the product of the number of person-years and the sex-, age-, and calendar-year specific South Korean thyroid cancer incidence rates (http://www.ncc.re.kr/). Reference rates were limited to the follow-up period 1999–2015 because nationwide site- and age-specific cancer incidence rates were only available from 1999 in South Korea. For the period 1996–1998, we assumed that the cancer incidence rates were the same as those for 1999. Our analyses focused on the first primary thyroid cancer.

Standardized incidence ratios (SIRs) and corresponding 95% confidence intervals (CIs) for thyroid cancers were calculated using the South Korean cancer incidence rates using Poisson-regression methods using the AMFIT module in Epicure [23]. Relative risks (RRs) were calculated by Poisson regression adjusted for attained age, sex, and calendar year. The trends of SIRs and RRs with dose categories were investigated by using simple dose response models.

The radiation effect on thyroid cancer rates was described using a linear ERR dose-response model. This model can be written as: *λ*(*a*, *g*, *t*, *d*) = *λ*_0_(*a*, *g*, *t*)[1 + *δd*] where *a*, *g*, *t*, and *d* denote attained age, sex, calendar time, and occupational radiation dose, respectively. Background cancer incidence rate was described as *λ*_0_(*a*, *g*, *t*) depending on attained age, sex, and calendar time. The model was parameterized so that δ was an estimate of the ERR per 100 mGy. Parameter estimates and 95% confidence bounds were obtained using the maximum likelihood method using the AMFIT module in Epicure. Linear-quadratic and quadratic terms did not improve the model fit. Sensitivity analyses were conducted by excluding workers who worked for < 1 year and by consideration of unlagged, five-year lagged and ten-year lagged cumulative doses. We also examined variation in baseline rates and radiation risks by job title (physicians and non-physicians).

## Results

A total of 827 cancer cases (309 for men, 518 for women) were identified among all diagnostic medical radiation workers during the study period in South Korea, yielding 1,101,448 person years (Table 1). Age-adjusted thyroid cancer rates were higher for women, those who began work in earlier years, and those with a longer duration of work. The majority of workers were born after 1960 and more than 70% of cases were diagnosed between the ages of 20 and 40 years. Cumulative thyroid doses were similar for cases and non-cases. Papillary cancer was the predominant historical type (96.5%), and the majority of cases (86.9%) were regional or localized stage tumors.

**Table 1:** Characteristics of thyroid cancer cases and non-cases at baseline among South Korean medical radiation workers, 1996–2015

	Thyroid cancer cases (*n* = 827)	Thyroid non-cases (*n* = 93,095)
	Numbers (%)	Numbers (%)
Person-years at risk	6772	1,094,676
Sex		
Male	309 (37.4)	53,276 (57.2)
Female	518 (62.6)	39,819 (42.8)
Age at baseline, years		
< 25	167 (20.2)	20,785 (22.3)
25–29	227 (27.5)	26,594 (28.6)
30–39	295 (35.7)	29,776 (32.0)
≥ 40	138 (16.7)	15,940 (17.1)
Calendar year of birth		
< 1960	94 (11.4)	10,403 (11.2)
1960–1969	259 (31.3)	24,085 (25.9)
1970–1979	321 (38.8)	32,576 (35.0)
≥ 1980	153 (18.5)	26,031 (28.0)
Calendar year of entry		
1996–1999	280 (33.9)	23,066 (24.8)
2000–2004	239 (28.9)	24,689 (26.5)
2005–2011	308 (37.2)	45,340 (48.7)
Type of medical facility		
Hospital	291 (35.2)	31,375 (33.7)
Clinic	229 (27.7)	25,906 (27.8)
Others	307 (37.1)	35,814 (38.5)
Duration of employment, years		
< 1	90 (10.9)	13,349 (14.3)
1–4	294 (35.6)	36,449 (39.2)
5–9	210 (25.4)	22,517 (24.2)
≥ 10	233 (28.2)	20,780 (22.3)
Cumulative thyroid dose (mGy),		
Mean ± SD	9.9 ± 32.3	10.4 ± 41.6
Median (range)	1.5 (0.007, 579.2)	1.4 (0.007, 9861.2)
< 0.5	245 (29.6)	29,970 (32.2)
0.5–1.0	100 (12.1)	11,515 (12.4)
1.0–2.5	138 (16.7)	14,393 (15.5)
2.5–5.0	89 (10.8)	8710 (9.4)
5.0–10.0	66 (8.0)	8296 (8.9)
10.0–20.0	85 (10.3)	7265 (7.8)
≥ 20.0	104 (12.6)	12,946 (13.9)
Age at diagnosis, years		
< 30	112 (13.5)	–
30–39	309 (37.4)	–
40–49	292 (35.3)	–
≥ 50	114 (13.8)	–
Histological type		
Papillary	798 (96.5)	–
Follicular	9 (1.1)	–
Medullary	5 (0.6)	–
Others	15 (1.8)	–
SEER summary stage		
Localized	311 (37.6)	–
Regional	408 (49.3)	–
Distant	5 (0.7)	–
Unknown/missing	103 (12.4)	–

*SD* standard deviation, *SEER* Surveillance, Epidemiology and End Results

**Fig. 1:**
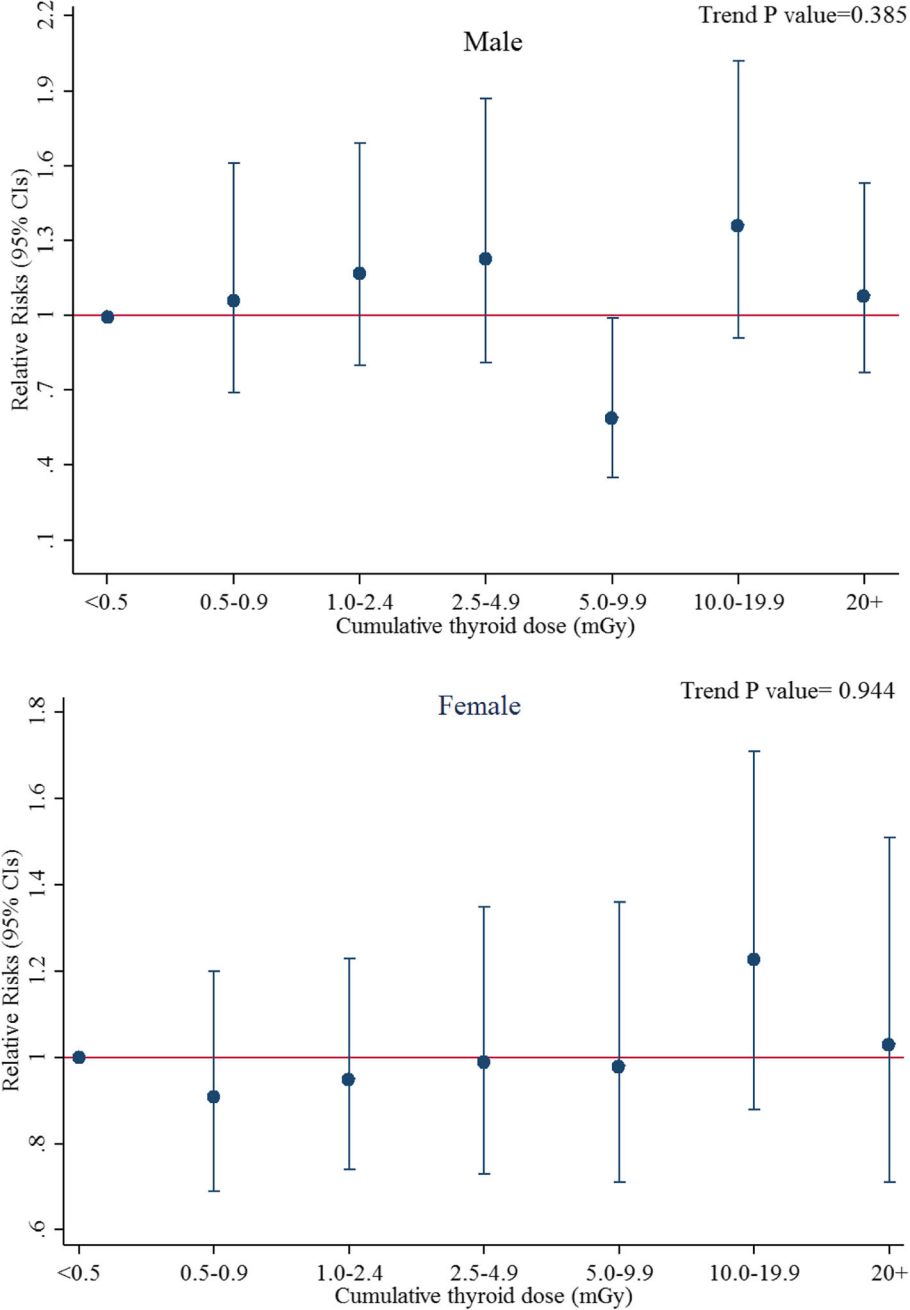
Relative risks for thyroid cancer by cumulative thyroid dose categories stratified by sex among South Korean medical radiation workers, 1996–2015. Relative risks were adjusted for attained age (< 25, 5-year intervals from age 25 to 84, ≥85 years) and calendar time (< 2000, 2000–2004, 2005–2009, ≥2010)

SIR analysis of thyroid cancer revealed significantly increased incidence relative to national rates for both men (SIR 1.72, 95% CI 1.53–1.91) and women (SIR 1.18, 95% CI 1.08–1.28) (Table 2). The observed SIRs were similar within job characteristic categories, while men had larger SIRs than women (*p* < 0.001). The RR trend provided no evidence of a dose-response relationship with cumulative thyroid dose categories (Fig. 1). Similar risk estimates were seen when the analyses were limited to the workers who had been employed for at least one year (Additional file 1: Table S1).

**Table 2:** Standardized incidence ratios and relative risks for thyroid cancer by occupational history and sex among South Korean medical radiation workers, 1996–2015

	Male			Female		
	Cases	SIR (95% CI)	RR^a^ (95% CI)	Cases	SIR (95% CI)	RR^a^ (95% CI)
Total	309	1.72 (1.53, 1.91)	–	518	1.18 (1.08, 1.28)	–
Job title						
Radiologic technologist	91	1.73 (1.37, 2.08)	Ref (1.00)	141	1.60 (1.34, 1.87)	Ref (1.00)
Radiologist	11	2.33 (0.95, 3.71)	1.42 (0.75, 2.69)	10	1.07 (0.41, 1.73)	0.63 (0.33, 1.22)
Dentist	68	1.60 (1.22, 1.98)	1.06 (0.76, 1.47)	51	1.05 (0.76, 1.34)	0.65 (0.47, 0.91)
Dental hygienist	0	–	–	101	0.88 (0.71, 1.05)	0.59 (0.45, 0.76)
Nurse	2	2.24 (−, 5.35)	1.34 (0.33, 5.44)	80	1.17 (0.91, 1.43)	0.77 (0.58, 1.01)
Doctor	97	1.73 (1.38, 2.07)	1.11 (0.81, 1.51)	53	1.48 (1.08, 1.88)	0.93 (0.67, 1.30)
Others	40	1.79 (1.23, 2.34)	0.98 (0.67, 1.43)	82	1.11 (0.87, 1.35)	0.69 (0.52, 0.91)
Type of medical facility						
Hospital	101	1.81 (1.46, 2.16)	Ref (1.00)	190	1.42 (1.21, 1.62)	Ref (1.00)
Clinic	118	1.72 (1.41, 2.02)	1.01 (0.77, 1.34)	111	1.30 (1.06, 1.54)	0.91 (0.72, 1.15)
Others	90	1.65 (1.31, 1.98)	1.02 (0.76, 1.37)	217	0.99 (0.86, 1.12)	0.72 (0.59, 0.87)
Year of birth						
< 1960	68	1.88 (1.43, 2.32)	1.25 (0.46, 3.35)	26	1.45 (0.89, 2.01)	2.01 (0.89, 4.56)
1960–1969	131	1.77 (1.47, 2.08)	1.17 (0.54, 2.51)	128	1.25 (1.03, 1.47)	1.10 (0.66, 1.84)
1970–1979	77	1.36 (1.06, 1.66)	0.94 (0.53, 1.68)	244	1.17 (1.03, 1.32)	1.15 (0.83, 1.60)
≥ 1980	33	2.60 (1.71, 3.49)	Ref (1.00)	120	1.09 (0.89, 1.28)	Ref (1.00)
Year of entry						
1996–1999	126	1.85 (1.53, 2.18)	0.93 (0.69, 1.24)	154	1.32 (1.11, 1.53)	1.11 (0.87, 1.41)
2000–2004	77	1.42 (1.10, 1.73)	0.73 (0.54, 0.98)	162	1.19 (1.01, 1.37)	1.06 (0.86, 1.32)
2005–2011	106	1.86 (1.50, 2.21)	Ref (1.00)	202	1.09 (0.94, 1.24)	Ref (1.00)
Age at baseline, years						
< 25	18	1.75 (0.94, 2.56)	Ref (1.00)	149	1.15 (0.97, 1.34)	Ref (1.00)
25–29	70	1.69 (1.30, 2.09)	1.66 (0.90, 3.05)	157	1.19 (1.00, 1.38)	1.10 (0.86, 1.40)
30–39	136	1.73 (1.44, 2.02)	2.06 (1.06, 4.00)	159	1.17 (0.99, 1.35)	1.00 (0.75, 1.35)
≥ 40	85	1.73 (1.36, 2.10)	1.90 (0.90, 4.02)	53	1.30 (0.95, 1.64)	1.20 (0.77, 1.88)
Duration of employment, years						
< 1	18	1.36 (0.73, 1.99)	Ref (1.00)	72	1.19 (0.92, 1.47)	Ref (1.00)
1–4	90	1.90 (1.51, 2.29)	1.41 (0.85, 2.35)	204	1.08 (0.93, 1.22)	0.90 (0.69, 1.18)
5–9	81	1.58 (1.23, 1.92)	1.15 (0.68, 1.93)	129	1.17 (0.97, 1.37)	0.94 (0.70, 1.26)
≥ 10	120	1.78 (1.46, 2.10)	1.25 (0.75, 2.10)	113	1.44 (1.18, 1.71)	1.11 (0.81, 1.53)

^a^Adjusted for attained age (< 25, 5-year intervals from age 25 to 84, ≥85 years) and calendar time (< 2000, 2000–2004, 2005–2009, ≥2010)

*SIR* standardized incidence ratio, *CI* confidence interval, *Ref*. reference, *RR* relative risk

There was no evidence of a statistically significant cumulative occupational radiation dose-response relationship with thyroid cancer risk in either men (ERR/100 mGy 0.07) or women (ERR/100 mGy − 0.13) (Table 3). The risks were not significantly different by sex, job title, year of birth, or work duration. The findings of a sensitivity analysis limited to workers who were employed for at least one year were similar. The results, based on unlagged or 10-year lagged doses were similar to those from the 5-year lagged dose analyses (Additional files 2 and 3: Table S2 and S3). Stratified analyses of RRs and ERRs by job title (physicians and non-physicians) that are likely to reflect differences in some lifestyle factors showed no particular pattern of thyroid cancer risks (Additional file 4: Table S4).

**Table 3:** Excess relative risks per 100 mGy for thyroid cancer with 5-year lag by occupational history among South Korean medical radiation workers, 1996–2015

	All		Employment duration ≥1 year	
	Cases	ERR^a^ per 100 mGy (95% CI)	Cases	ERR^a^ per 100 mGy (95% CI)
Overall	827	0.04 (−0.35, 0.43)	737	0.03 (−0.35, 0.42)
Sex				
Male	309	0.07 (−0.38, 0.53)	291	0.06 (−0.39, 0.52)
Female	518	−0.13 (−0.49, 0.23)	446	-0.13 (−0.68, 0.43)
Job title				
Radiologic technologist	232	0.01 (−0.49, 0.52)	218	0.01 (−0.50, 0.51)
Radiologist	21	0.01 (−0.84, 0.85)	21	0.002 (−0.84, 0.84)
Dentist	119	−0.05 (−4.57, 4.48)	115	−0.06 (−4.57, 4.45)
Dental hygienist	101	−1.16 (−4.53, 2.21)	82	−1.14 (−5.22, 2.94)
Nurse	82	−0.11 (−1.87, 1.65)	61	−0.11 (−1.85, 1.64)
Doctor	150	−0.003 (−1.20, 1.19)	141	−0.01 (−1.20, 1.19)
Others	122	0.03 (−0.69, 0.74)	99	0.03 (−0.69, 0.75)
Type of medical facility				
Hospital	291	0.02 (−0.47, 0.50)	251	−0.004 (−0.48, 0.48)
Clinic	229	0.004 (−0.67, 0.68)	218	−0.01 (−0.68, 0.66)
Others	307	0.07 (−0.76, 0.91)	268	0.09 (−0.74, 0.93)
Year of birth				
< 1960	94	0.004 (−0.46, 0.47)	91	0.004 (−0.47, 0.47)
1960–1969	259	0.001 (−0.73, 0.73)	248	−0.002 (−0.73, 0.72)
1970–1979	321	0.03 (−0.95, 1.01)	279	0.03 (−0.96, 1.03)
≥ 1980	153	−0.16 (−2.40, 2.08)	119	−0.16 (−2.46, 2.15)
Year of entry				
1996–1999	280	0.03 (−0.37, 0.42)	274	0.02 (−0.37, 0.42)
2000–2004	239	−0.02 (−1.28, 1.23)	218	−0.001 (−1.29, 1.28)
2005–2011	308	−0.86 (−3.42, 1.71)	245	−0.87 (−3.45, 1.72)
Age at baseline, years				
< 25	167	0.14 (−1.38, 1.65)	149	0.16 (−1.40, 1.71)
25–29	227	−0.10 (−0.88, 0.69)	195	−0.10 (−0.88, 0.69)
30–39	295	−0.02 (−0.71, 0.67)	264	−0.03 (−0.71, 0.66)
≥ 40	138	0.04 (−0.49, 0.57)	129	0.04 (−0.49, 0.57)
Duration of employment, years				
< 1	90	−0.45 (−7.12, 6.23)	0	–
1–4	294	−0.08 (−2.12, 1.96)	294	−0.18 (−1.64, 1.28)
5–9	210	−0.07 (−1.44, 1.30)	210	−0.17 (−1.44, 1.11)
≥ 10	233	0.02 (−0.38, 0.41)	233	0.03 (−0.37, 0.43)

^a^Adjusted for sex, attained age (< 25, 5-year intervals from age 25 to 84, ≥85 years) and calendar time (< 2000, 2000–2004, 2005–2009, ≥2010)

*CI* confidence interval, *ERR* excess relative risk

## Discussion

Although thyroid cancer incidence rates were significantly greater than expected based on population rates among South Korean medical radiation workers, the RRs with the association of occupational factors and the ERR with cumulative radiation doses after adjusting for age, sex, and calendar year did not provide any evidence of a statistically significant effect on thyroid cancer risk. Findings were similar for the analysis limited to workers who were employed for at least one year or using unlagged or 10-year lagged cumulative doses. Our findings suggest that the higher SIRs observed in medical radiation workers may be a consequence of non-occupational factors although this should be reconsidered using longer follow-up and more detailed information on lifestyle and environmental exposures, especially during childhood.

The finding of increased thyroid cancer incidence among medical radiation workers relative to the general population SIRs is consistent with the findings of medical worker studies among US radiologic technologists [24], Canadian radiation workers [25], and South Korean radiologic technologists [26]. Our finding of no significant associations with occupational factors was consistent with that of Norwegian nurses [27], while the US radiologic technologists [28] and Chinese X-ray workers [29] reported some evidence of a potential occupational etiology of thyroid cancer. However, a recent study on US radiologic technologists found no association between cumulative occupational radiation doses and thyroid cancer risk, after adjusting for potential risk factors [30]. The overall pattern of thyroid cancer risks in this study (i.e., increased risk based on external comparison but no association with work history and radiation doses based on internal analyses), is generally consistent with the series of overall thyroid cancer findings of the US radiologic technologists study [24, 28, 30]. Similarly, a cohort study of nuclear power plant workers [31] and a female workers study from the national health insurance data [32] in South Korea reported increased thyroid cancer risks compared with the general population, but such increases disappeared when compared with other workers.

These findings generally support the hypothesis that the increased thyroid cancer incidence observed among Korean medical radiation workers may be explained by a diagnostic screening bias that arises from the differential access to health care. Radiation workers in South Korea may have greater surveillance as a consequence of legally mandated special annual medical examinations [33]. Although they are not specifically designed to screen for thyroid cancer, these examinations may facilitate detection. An age-period-cohort analysis also suggested the role of cancer screening in the rapid increase in thyroid cancer between 1997 and 2011 in South Korea [34], and many subjects in our study could be affected by this period effect.

However, the increased thyroid cancer risk may not be fully explained solely by the easier access to medical care by medical radiation workers because screening itself does not cause thyroid cancer. True increases in thyroid cancer in the US [35] and worldwide [36] have been reported, and changes in the prevalence of environmental risk factors were suggested as possible risk factors for this increasing incidence in thyroid cancer [5]. There are also some reports that certain occupations are associated with an increased risk of thyroid cancer [10, 37]. Therefore, more research is needed to identify potential thyroid cancer incidence risk factors, including lifestyle factors such as obesity, smoking, and diet; predisposing medical conditions; reproductive and hormonal factors; and environmental factors [38]. It could be difficult to find an association between radiation exposure and thyroid cancer in this low-dose adult-exposed cohort; therefore, medical and environmental radiation exposure among children or adolescents should receive special attention because of the marked and persistent radiation effects of such exposures on thyroid cancer rates [39].

Our study has several limitations that reduce the power to detect radiation effects on thyroid cancer risks. First, due to the short follow-up and strict radiation protection standards, the individual cumulative radiation exposures in this cohort (mean dose: 10.4 mGy, 90 percentile: 29.4 mGy) are considerably lower than the doses in other studies of medical workers such as the US radiologic technologists (mean: 57 mGy) [30] and Chinese medical workers (employed before 1970: 551 mGy, others: 82 mGy) [29]. Further studies focusing on relatively high-exposure medical workers, such as those performing fluoroscopically guided procedures may shed more light on the radiation and the risk of thyroid cancer. Second, the relatively young cohort (mean age at baseline: 32 years) and short follow-up (mean: 11.7 years) are likely to have limited our ability to detect radiation-induced cases because this cohort had not yet reached cancer prone ages. Third, other potential risk factors for thyroid cancer that could modulate or confound the findings could not be examined in this cohort; however, these were unlikely to confound the thyroid cancer risk from radiation exposure in this cohort.

This study has several strengths. First, the study population included all diagnostic medical radiation workers in South Korea making it possible to consider all diagnostic medical worker job categories. Second, among studies of medical radiation workers this study has the largest number of thyroid cancer cases and ascertainment was based on a comprehensive nationwide cancer registry system. Third, radiation effect estimates were based on thyroid doses constructed from individual badge dose readings while previous studies used exposure information abstracted from a combination of data from occupational records and badge dosimeters or simulated badge readings based on work history characteristics.

## Conclusions

In summary, even though South Korean medical radiation workers have markedly higher thyroid cancer rates than the general population, we found no evidence of a statistically significant association between thyroid dose from occupational radiation exposure and thyroid cancer rates in this comprehensive nationwide cohort. Our findings contribute to a better understanding of the observed thyroid cancer risk in adults. Future studies in this cohort with additional follow-up, information on screening history, radiation exposure during childhood, and other potential risk factors may help clarify the role of environmental and occupational risk factors in the development of thyroid cancer.

## Additional files

Additional file 1: **Table S1.** Standardized incidence ratios and relative risks for thyroid cancer by occupational history stratified by sex among South Korean medical radiation workers employed for at least one year during 1996–2015. (DOCX 20 kb)

Additional file 2: **Table S2.** Excess relative risks per 100 mGy for thyroid cancer without lag by occupational history among South Korean medical radiation workers, 1996–2015. (DOCX 17 kb)

Additional file 3: **Table S3.** Excess relative risks per 100 mGy for thyroid cancer with 10-year lag by occupational history among South Korean medical radiation workers, 1996–2015. (DOCX 17 kb)

Additional file 4: **Table S4.** Relative risks and excess relative risks for thyroid cancer by occupational history stratified by job title (physicians and non-physicians) among South Korean medical radiation workers, 1996–2015. (DOCX 17 kb)

## Abbreviations

CI: Confidence interval
ERR: Excess relative risk
KCDC: Korea Centers for Disease Control and Prevention
NDR: National Dosimetry Registry
Ref: Reference
RR: Relative risk
SD: Standard deviation
SEER: Surveillance, Epidemiology and End Results
SIR: Standardized incidence ratios
