# Supplementary material for: Thyroid cancer risks among medical radiation workers in South Korea, 1996–2015
Source: Environ Health. 2019 Mar 11;18:19. doi: 10.1186/s12940-019-0460-z (PMC6413450; doi:10.1186/s12940-019-0460-z)
Supplement: Supplementary file 1 — Table S1. Standardized incidence ratios and relative risks for thyroid cancer by occupational history stratified by sex among South Korean medical radiation workers employed for at least one year during 1996–2015. (DOCX 20 kb) [file 12940_2019_460_MOESM1_ESM.docx]

Table S1. Standardized incidence ratios and relative risks for thyroid cancer by occupational history stratified by sex among South Korean medical radiation workers employed for at least one year during 1996–2015

|  | Male | | | |  | Female | | |
| --- | --- | --- | --- | --- | --- | --- | --- | --- |
|  | Cases | | SIR (95% CI) | RR^a^ (95% CI) |  | Cases | SIR (95% CI) | RR^a^ (95% CI) |
| Total | 291 | | 1.75 (1.56, 1.96) | - |  | 446 | 1.18 (1.08, 1.29) | - |
| Job title |  | |  |  |  |  |  |  |
| Radiologic technologist | 86 | | 1.68 (1.33, 2.04) | Ref (1.00) |  | 132 | 1.56 (1.30, 1.83) | Ref (1.00) |
| Radiologist | 11 | | 2.35 (0.96, 3.73) | 1.41 (0.75, 2.67) |  | 10 | 1.07 (0.41, 1.73) | 0.64 (0.33, 1.24) |
| Dentist | 65 | | 1.60 (1.21, 1.99) | 1.04 (0.74, 1.46) |  | 50 | 1.09 (0.79, 1.40) | 0.69 (0.49, 0.97) |
| Dental hygienist | 0 | | - | - |  | 82 | 0.85 (0.66, 1.03) | 0.58 (0.44, 0.76) |
| Nurse | 2 | | 2.97 (-, 7.08) | 1.87 (0.46, 7.60) |  | 59 | 1.08 (0.81, 1.36) | 0.72 (0.53, 0.98) |
| Doctor | 92 | | 1.78 (1.41, 2.14) | 1.11 (0.81, 1.53) |  | 49 | 1.50 (1.08, 1.93) | 0.95 (0.68, 1.35) |
| Others | 35 | | 2.03 (1.36, 2.70) | 1.11 (0.74, 1.65) |  | 64 | 1.17 (0.88, 1.45) | 0.72 (0.53, 0.98) |
| Type of medical facility |  | |  |  |  |  |  |  |
| Hospital | 94 | | 1.84 (1.47, 2.21) | Ref (1.00) |  | 157 | 1.34 (1.13, 1.55) | Ref (1.00) |
| Clinic | 113 | | 1.75 (1.43, 2.08) | 0.99 (0.75, 1.32) |  | 105 | 1.35 (1.09, 1.60) | 0.99 (0.78, 1.27) |
| Others | 84 | | 1.66 (1.30, 2.01) | 0.98 (0.72, 1.34) |  | 184 | 1.00 (0.86, 1.15) | 0.77 (0.62, 0.95) |
| Year of birth |  | |  |  |  |  |  |  |
| <1960 | 65 | | 1.88 (1.42, 2.33) | 1.05 (0.37, 2.94) |  | 26 | 1.55 (0.95, 2.15) | 2.28 (0.96, 5.39) |
| 1960 - 1969 | 131 | | 1.86 (1.54, 2.17) | 1.06 (0.47, 2.41) |  | 117 | 1.24 (1.02, 1.47) | 1.12 (0.64, 1.93) |
| 1970 - 1979 | 70 | | 1.37 (1.05, 1.70) | 0.92 (0.49, 1.73) |  | 209 | 1.17 (1.01, 1.33) | 1.16 (0.81, 1.66) |
| ≥1980 | 25 | | 2.48 (1.51, 3.46) | Ref (1.00) |  | 94 | 1.06 (0.85, 1.28) | Ref (1.00) |
| Year of entry |  | |  |  |  |  |  |  |
| 1996-1999 | 126 | | 1.89 (1.56, 2.22) | 0.90 (0.67, 1.21) |  | 148 | 1.34 (1.12, 1.55) | 1.18 (0.91, 1.53) |
| 2000-2004 | 73 | | 1.44 (1.11, 1.78) | 0.72 (0.52, 0.99) |  | 145 | 1.21 (1.01, 1.40) | 1.13 (0.89, 1.43) |
| 2005-2011 | 92 | | 1.88 (1.49, 2.26) | Ref (1.00) |  | 153 | 1.04 (0.87, 1.20) | Ref (1.00) |
| Age at baseline, years |  | |  |  |  |  |  |  |
| <25 | 16 | | 1.87 (0.96, 2.79) | Ref (1.00) |  | 133 | 1.16 (0.96, 1.36) | Ref (1.00) |
| 25-29 | 62 | | 1.60 (1.20, 2.00) | 1.45 (0.75, 2.81) |  | 133 | 1.20 (0.99, 1.40) | 1.04 (0.81, 1.35) |
| 30-39 | 131 | | 1.78 (1.48, 2.09) | 1.78 (0.87, 3.64) |  | 133 | 1.13 (0.94, 1.32) | 0.89 (0.64, 1.22) |
| ≥40 | 82 | | 1.80 (1.41, 2.20) | 1.71 (0.78, 3.79) |  | 47 | 1.34 (0.96, 1.73) | 1.13 (0.70, 1.83) |
| Duration of employment, years | | |  |  |  |  |  |  |
| 1-4 | 90 | | 1.90 (1.51, 2.29) | Ref (1.00) |  | 204 | 1.08 (0.93, 1.22) | Ref (1.00) |
| 5-9 | 81 | | 1.58 (1.23, 1.92) | 0.79 (0.58, 1.07) |  | 129 | 1.17 (0.97, 1.37) | 1.04 (0.83, 1.30) |
| ≥10 | 120 | | 1.78 (1.46, 2.10) | 0.84 (0.62, 1.13) |  | 113 | 1.44 (1.18, 1.71) | 1.22 (0.95, 1.58) |
| Cumulative thyroid dose, mSv | | |  |  |  |  |  |  |
| <0.5 | | 52 | 1.73 (1.26, 2.20) | Ref (1.00) |  | 124 | 1.17 (0.96, 1.37) | Ref (1.00) |
| 0.5-1.0 | | 30 | 1.66 (1.06, 2.25) | 0.93 (0.59, 1.46) |  | 62 | 1.07 (0.80, 1.34) | 0.89 (0.66, 1.21) |
| 1.0-2.5 | | 49 | 1.90 (1.37, 2.43) | 1.07 (0.72, 1.58) |  | 83 | 1.12 (0.88, 1.36) | 0.92 (0.69, 1.21) |
| 2.5-5.0 | | 33 | 1.96 (1.29, 2.63) | 1.08 (0.70, 1.68) |  | 52 | 1.18 (0.86, 1.50) | 0.96 (0.69, 1.32) |
| 5.0-10.0 | | 18 | 0.99 (0.53, 1.45) | 0.54 (0.32, 0.93) |  | 46 | 1.19 (0.84, 1.53) | 0.95 (0.67, 1.34) |
| 10.0-20.0 | | 39 | 2.23 (1.53, 2.93) | 1.23 (0.81, 1.87) |  | 45 | 1.53 (1.09, 1.98) | 1.21 (0.86, 1.72) |
| ≥20.0 | | 70 | 1.76 (1.35, 2.18) | 0.99 (0.69, 1.42) |  | 34 | 1.25 (0.83, 1.67) | 1.01 (0.68, 1.50) |

^a^Adjusted for attained age (<25, 5-year intervals from age 25 to 84, ≥85 years) and calendar time (<2000, 2000-2004, 2005-2009, ≥2010)

SIR, standardized incidence ratio; CI, confidence interval; RR, relative risk; Ref., reference
