# Supplementary material for: Thyroid cancer risks among medical radiation workers in South Korea, 1996–2015
Source: Environ Health. 2019 Mar 11;18:19. doi: 10.1186/s12940-019-0460-z (PMC6413450; doi:10.1186/s12940-019-0460-z)
Supplement: Supplementary file 2 — Table S2. Excess relative risks per 100 mGy for thyroid cancer without lag by occupational history among South Korean medical radiation workers, 1996–2015. (DOCX 17 kb) [file 12940_2019_460_MOESM2_ESM.docx]

Table S2. Excess relative risks per 100 mGy for thyroid cancer without lag by occupational history among South Korean medical radiation workers, 1996-2015

|  | All | | Employment duration ≥1 year | |
| --- | --- | --- | --- | --- |
|  | Cases | ERR^a^ per 100 mGy (95% CI) | Cases | ERR^a^ per 100 mGy (95% CI) |
| Overall | 827 | 0.08 (-0.29, 0.45) | 737 | 0.06 (-0.30, 0.43) |
| Sex |  |  |  |  |
| Male | 309 | 0.08 (-0.35, 0.51) | 291 | 0.07 (-0.36, 0.50) |
| Female | 518 | 0.05 (-0.64, 0.74) | 446 | 0.01 (-0.66, 0.68) |
| Job title |  |  |  |  |
| Radiologic technologist | 232 | 0.02 (-0.43, 0.48) | 218 | 0.02 (-0.44, 0.48) |
| Radiologist | 21 | 0.01 (-0.81, 0.83) | 21 | 0.01 (-0.81, 0.82) |
| Dentist | 119 | -0.02 (-3.59, 3.54) | 115 | -0.04 (-3.60, 3.51) |
| Dental hygienist | 101 | -1.05 (-5.53, 3.44) | 82 | -1.03 (-5.75, 3.71) |
| Nurse | 82 | -0.12 (-1.69, 1.46) | 61 | -0.12 (-1.69, 1.44) |
| Doctor | 150 | -0.02 (-1.06, 1.02) | 141 | -0.02 (-1.07, 1.02) |
| Others | 122 | 0.04 (-0.67, 0.74) | 99 | 0.03 (-0.68, 0.75) |
| Type of medical facility |  |  |  |  |
| Hospital | 291 | 0.08 (-0.34, 0.50) | 251 | 0.06 (-0.35, 0.48) |
| Clinic | 229 | 0.05 (-0.58, 0.67) | 218 | 0.03 (-0.59, 0.65) |
| Others | 307 | -0.13 (-0.39, 0.12) | 268 | -0.13 (-0.31, 0.05) |
| Year of birth |  |  |  |  |
| <1960 | 94 | 0.03 (-0.44, 0.50) | 91 | 0.02 (-0.45, 0.48) |
| 1960 - 1969 | 259 | 0.02 (-0.64, 0.67) | 248 | -0.05 (-0.68, 0.58) |
| 1970 - 1979 | 321 | 0.46 (-0.47, 1.38) | 279 | 0.51 (-0.45, 1.46) |
| ≥1980 | 153 | 0.08 (-1.77, 1.93) | 119 | 0.003 (-1.85, 1.85) |
| Year of entry |  |  |  |  |
| 1996-1999 | 280 | 0.07 (-0.32, 0.45) | 274 | 0.07 (-0.32, 0.45) |
| 2000-2004 | 239 | -0.12 (-0.92, 0.67) | 218 | -0.12 (-0.80, 0.56) |
| 2005-2011 | 308 | -0.11 (-1.87, 1.65) | 245 | -0.27 (-2.00, 1.47) |
| Age at baseline, years |  |  |  |  |
| <25 | 167 | 0.33 (-0.95, 1.61) | 149 | 0.34 (-0.97, 1.64) |
| 25-29 | 227 | -0.13 (-0.50, 0.24) | 195 | -0.13 (-0.50, 0.24) |
| 30-39 | 295 | -0.03 (-0.65, 0.59) | 264 | -0.04 (-0.65, 0.57) |
| ≥40 | 138 | 0.08 (-0.46, 0.62) | 129 | 0.07 (-0.47, 0.61) |
| Duration of employment, years |  |  |  |  |
| <1 | 90 | 0.76 (-8.29, 9.81) | 0 | - |
| 1-4 | 294 | 0.19 (-1.65, 2.03) | 294 | 0.21 (-1.67, 2.08) |
| 5-9 | 210 | -0.12 (-1.08, 0.84) | 210 | -0.12 (-1.10, 0.86) |
| ≥10 | 233 | 0.04 (-0.34, 0.41) | 233 | 0.03 (-0.35, 0.41) |

^a^Adjusted for sex, attained age (<25, 5-year intervals from age 25 to 84, ≥85 years) and calendar time (<2000, 2000-2004, 2005-2009, ≥2010)

CI, confidence interval; ERR, excess relative risk
