# Supplementary material for: Thyroid cancer risks among medical radiation workers in South Korea, 1996–2015
Source: Environ Health. 2019 Mar 11;18:19. doi: 10.1186/s12940-019-0460-z (PMC6413450; doi:10.1186/s12940-019-0460-z)
Supplement: Supplementary file 3 — Table S3. Excess relative risks per 100 mGy for thyroid cancer with 10-year lag by occupational history among South Korean medical radiation workers, 1996–2015. (DOCX 17 kb) [file 12940_2019_460_MOESM3_ESM.docx]

Table S3. Excess relative risks per 100 mGy for thyroid cancer with 10-year lag by occupational history among South Korean medical radiation workers, 1996–2015

|  | All | | Employment duration ≥1 year | |
| --- | --- | --- | --- | --- |
|  | Cases | ERR^a^ per 100 mGy  (95% CI) | Cases | ERR^a^ per 100 mGy (95% CI) |
| Overall | 827 | -0.01 (-0.42, 0.40) | 737 | -0.02 (-0.42, 0.39) |
| Sex |  |  |  |  |
| Male | 309 | 0.03 (-0.45, 0.51) | 291 | 0.02 (-0.46, 0.50) |
| Female | 518 | -0.13 (-0.24, -0.03) | 446 | -0.11 (-0.75, 0.53) |
| Job title |  |  |  |  |
| Radiologic technologist | 232 | -0.01 (-0.61, 0.59) | 218 | -0.01 (-0.62, 0.58) |
| Radiologist | 21 | -0.02 (-0.88, 0.85) | 21 | -0.02 (-0.88, 0.83) |
| Dentist | 119 | 0.16 (-6.81, 7.13) | 115 | 0.17 (-6.79, 7.14) |
| Dental hygienist | 101 | -1.14 (-9.82, 7.55) | 82 | -1.29 (-3.27, 0.69) |
| Nurse | 82 | -0.08 (-2.18, 2.03) | 61 | -0.10 (-2.16, 1.96) |
| Doctor | 150 | -0.05 (-1.43, 1.34) | 141 | -0.06 (-1.44, 1.32) |
| Others | 122 | 0.01 (-1.59, 1.61) | 99 | 0.01 (-1.57, 1.59) |
| Type of medical facility |  |  |  |  |
| Hospital | 291 | 0.04 (-0.44, 0.52) | 251 | 0.03 (-0.44, 0.50) |
| Clinic | 229 | -0.21 (-0.56, 0.14) | 218 | -0.21 (-0.23, -0.19) |
| Others | 307 | -0.02 (-1.49, 1.44) | 268 | -0.03 (-1.47, 1.41) |
| Year of birth |  |  |  |  |
| <1960 | 94 | 0.01 (-0.47, 0.49) | 91 | 0.005 (-0.48, 0.49) |
| 1960 - 1969 | 259 | -0.05 (-0.89, 0.80) | 248 | -0.05 (-0.88, 0.78) |
| 1970 - 1979 | 321 | 0.05 (-1.40, 1.50) | 279 | 0.06 (-1.41, 1.54) |
| ≥1980 | 153 | -0.16 (-3.62, 3.30) | 119 | -0.15 (-3.84, 3.54) |
| Year of entry |  |  |  |  |
| 1996-1999 | 280 | -0.01 (-0.42, 0.41) | 274 | -0.01 (-0.42, 0.41) |
| 2000-2004 | 239 | -0.02 (-2.12, 2.08) | 218 | 0.14 (-2.18, 2.46) |
| 2005-2011 | 308 | -1.54 (-20.81, 17.72) | 245 | -0.79 (-22.35, 20.76) |
| Age at baseline, years |  |  |  |  |
| <25 | 167 | 0.17 (-2.18, 2.52) | 149 | 0.20 (-2.22, 2.62) |
| 25-29 | 227 | -0.11 (-1.00, 0.79) | 195 | -0.11 (-0.99, 0.78) |
| 30-39 | 295 | -0.07 (-0.87, 0.73) | 264 | -0.07 (-0.87, 0.72) |
| ≥40 | 138 | 0.03 (-0.50, 0.57) | 129 | 0.03 (-0.51, 0.57) |
| Duration of employment, years |  |  |  |  |
| <1 | 90 | -0.43 (-8.46, 7.60) | 0 | - |
| 1-4 | 294 | 0.04 (-3.38, 3.46) | 294 | 0.29 (-3.57, 4.15) |
| 5-9 | 210 | -0.02 (-2.23, 2.18) | 210 | -0.16 (-2.10, 1.79) |
| ≥10 | 233 | -0.001 (-0.42, 0.42) | 233 | -0.01 (-0.43, 0.41) |

^a^Adjusted for sex, attained age (<25, 5-year intervals from age 25 to 84, ≥85 years), calendar time (<2000, 2000-2004, 2005-2009, ≥2010)

CI, confidence interval; ERR, excess relative risk
