# Supplementary material for: Thyroid cancer risks among medical radiation workers in South Korea, 1996–2015
Source: Environ Health. 2019 Mar 11;18:19. doi: 10.1186/s12940-019-0460-z (PMC6413450; doi:10.1186/s12940-019-0460-z)
Supplement: Supplementary file 4 — Table S4. Relative risks and excess relative risks for thyroid cancer by occupational history stratified by job title (physicians and non-physicians) among South Korean medical radiation workers, 1996–2015. (DOCX 17 kb) [file 12940_2019_460_MOESM4_ESM.docx]

Table S4. Relative risks and excess relative risks for thyroid cancer by occupational history stratified by job title (physicians and non-physicians) among South Korean medical radiation workers, 1996–2015

|  | RR^a^ (95% CI) | |  | ERR^a^ per 100 mGy (95% CI) with 5-year lag | |
| --- | --- | --- | --- | --- | --- |
|  | Physicians | Non-physicians |  | Physicians | Non-physicians |
| Overall | - | - |  | -0.02  (-0.76, 0.72) | 0.10  (-0.48, 0.68) |
| Sex |  |  |  |  |  |
| Male | Ref (1.00) | Ref (1.00) |  | 0.16  (-0.94, 1.25) | 0.12  (-0.53, 0.78) |
| Female | 3.29  (2.57, 4.21) | 3.13  (2.54, 3.85) |  | -0.21  (-1.02, 0.60) | -0.11  (-0.97, 0.74) |
| Type of medical facility |  |  |  |  |  |
| Hospital | Ref (1.00) | Ref (1.00) |  | -0.03  (-0.85, 0.80) | 0.10  (-0.53, 0.73) |
| Clinic | 0.86  (0.62, 1.19) | 0.94  (0.76, 1.17) |  | 0.0004  (-1.37, 1.37) | 0.06  (-0.84, 0.96) |
| Others | 0.78  (0.57, 1.06) | 0.77  (0.63, 0.94) |  | -0.29  (-4.65, 4.07) | 0.55  (-1.55, 2.65) |
| Year of birth |  |  |  |  |  |
| <1960 | 1.17  (0.41, 3.36) | 1.11  (0.48, 2.56) |  | -0.06  (-0.84, 0.72) | 0.02  (-0.72, 0.75) |
| 1960 - 1969 | 0.82  (0.35, 1.90) | 1.19  (0.71, 1.98) |  | 0.22  (-1.70, 2.13) | 0.003  (-0.88, 0.88) |
| 1970 - 1979 | 0.67  (0.34, 1.34) | 1.26  (0.91, 1.73) |  | -0.72  (-4.56, 3.13) | 0.04  (-1.02, 1.09) |
| ≥1980 | Ref (1.00) | Ref (1.00) |  | 0.09  (-29.84, 30.03) | -0.16  (-2.51, 2.20) |
| Year of entry |  |  |  |  |  |
| 1996-1999 | 1.04  (0.76, 1.43) | 1.06  (0.82, 1.37) |  | 0.01  (-0.76, 0.78) | 0.11  (-0.51, 0.72) |
| 2000-2004 | 0.85  (0.64, 1.14) | 1.02  (0.81, 1.28) |  | -0.15  (-2.93, 2.64) | 0.03  (-1.42, 1.49) |
| 2005-2011 | Ref (1.00) | Ref (1.00) |  | -0.85  (-7.47, 5.77) | -1.58  (-2.66, -0.51) |
| Duration of employment, years |  |  |  |  |  |
| <1 | Ref (1.00) | Ref (1.00) |  | -1.96  (-34.41, 30.49) | -0.35  (-7.92, 7.21) |
| 1-4 | 1.55  (0.87, 2.77) | 0.88  (0.68, 1.15) |  | 0.65  (-7.27, 8.57) | -0.12  (-2.15, 1.92) |
| 5-9 | 1.00  (0.55, 1.80) | 1.08  (0.81, 1.44) |  | -0.16  (-3.50, 3.18) | -0.03  (-1.59, 1.53) |
| ≥10 | 1.25  (0.68, 2.28) | 1.08  (0.79, 1.47) |  | -0.0005  (-0.76, 0.76) | 0.03  (-0.54, 0.61) |

^a^Adjusted for sex, attained age (<25, 5-year intervals from age 25 to 84, ≥85 years) and calendar time (<2000, 2000-2004, 2005-2009, ≥2010)

RR, relative risk; CI, confidence interval; ERR, excess relative risk; Ref., reference
